# Comprehensive mapping of immune perturbations associated with severe COVID-19

**DOI:** 10.1126/sciimmunol.abd7114

**Published:** 2020-07-15

**Authors:** Leticia Kuri-Cervantes, M. Betina Pampena, Wenzhao Meng, Aaron M. Rosenfeld, Caroline A.G. Ittner, Ariel R. Weisman, Roseline S. Agyekum, Divij Mathew, Amy E. Baxter, Laura A. Vella, Oliva Kuthuru, Sokratis A. Apostolidis, Luanne Bershaw, Jeanette Dougherty, Allison R. Greenplate, Ajinkya Pattekar, Justin Kim, Nicholas Han, Sigrid Gouma, Madison E. Weirick, Claudia P. Arevalo, Marcus J. Bolton, Eileen C. Goodwin, Elizabeth M. Anderson, Scott E. Hensley, Tiffanie K. Jones, Nilam S. Mangalmurti, Eline T. Luning Prak, E. John Wherry, Nuala J. Meyer, Michael R. Betts

**Affiliations:** 1 Department of Microbiology, Perelman School of Medicine, University of Pennsylvania, Philadelphia, PA 19104, USA.; 2 Institute for Immunology, Perelman School of Medicine, University of Pennsylvania, Philadelphia, PA 19104, USA.; 3 Department of Pathology and Laboratory Medicine, Perelman School of Medicine, Philadelphia, PA19104, USA.; 4 Division of Pulmonary, Allergy and Critical Care, Center for Translational Lung Biology, Lung Biology Institute, Department of Medicine, Perelman School of Medicine, University of Pennsylvania, Philadelphia, PA, 19104, USA.; 5 Department of Systems Pharmacology and Translational Therapeutics, Perelman School of Medicine, University of Pennsylvania, Philadelphia, PA, 19104, USA.; 6 Division of Infectious Diseases, Department of Pediatrics, Children's Hospital of Philadelphia, Philadelphia, Pennsylvania, 19104, USA.; 7 Division of Rheumatology, Department of Medicine, Hospital of the University of Pennsylvania, Philadelphia, Pennsylvania, 19104, USA.; 8 Division of Gastroenterology, Department of Medicine, Hospital of the University of Pennsylvania, Philadelphia, Pennsylvania, 19104, USA.; 9 Parker Institute for Cancer Immunotherapy at the University of Pennsylvania, Philadelphia, Pennsylvania, 19104, USA.

## Abstract

Although critical illness has been associated with SARS-CoV-2-induced hyperinflammation, the immune correlates of severe COVID-19 remain unclear. Here, we comprehensively analyzed peripheral blood immune perturbations in 42 SARS-CoV-2 infected and recovered individuals. We identified extensive induction and activation of multiple immune lineages, including T cell activation, oligoclonal plasmablast expansion, and Fc and trafficking receptor modulation on innate lymphocytes and granulocytes, that distinguished severe COVID-19 cases from healthy donors or SARS-CoV-2-recovered or moderate severity patients. We found the neutrophil to lymphocyte ratio to be a prognostic biomarker of disease severity and organ failure. Our findings demonstrate broad innate and adaptive leukocyte perturbations that distinguish dysregulated host responses in severe SARS-CoV-2 infection and warrant therapeutic investigation.

## Teaser

Profound plasmablast expansion, innate cell modulation, and T cell activation are defining features of severe COVID-19.

## INTRODUCTION

The coronavirus-19-disease (COVID-19) pandemic caused by the severe acute respiratory syndrome coronavirus 2 (SARS-CoV-2) has surpassed 11 million cases world-wide, causing more than 500,000 deaths in 216 countries (*1*). While asymptomatic in some, SARS-CoV-2 infection can cause viral pneumonia that progresses to acute respiratory distress syndrome (ARDS), and even multi-organ failure, in severe cases (*2*, *3*). It is unclear whether disease severity is caused by the viral infection, the host response, or both, emphasizing the urgent need to understand the immune perturbations induced by SARS-CoV-2 (*3*). Knowledge of the immunological signatures of severe COVID-19 is continually evolving. Although lymphopenia has been linked to disease severity, the majority of published studies are based on retrospective analyses of clinical data (*3*–*9*).

Immune profiling studies to date have been conducted as single case reports or focused only on moderate, severe or recovered COVID-19 with limited numbers of individuals (*10*–*14*), and have not necessarily reflected the range of comorbidities globally associated with severe COVID-19. Studies of peripheral blood mononuclear cells by mass cytometry or single cell RNA sequencing (scRNAseq) have provided valuable insights into possible immune perturbations in COVID-19 but have not assessed the contributions of granulocytic populations, or, in the case of scRNAseq, defined expression or modulation of cellular proteins (*11*). In particular, modulation of granulocytic populations is suggested to be relevant during COVID-19 infection (*15*).

To address these issues, we conducted a comprehensive analysis of the overall immunologic state of 42 individuals with different trajectories of SARS-CoV-2 infection and COVID-19 (moderate, severe, and recovered), compared with 12 healthy donors (HD) using whole blood to capture the full breadth of immunological perturbations and activation occurring in circulating lymphocytes and major granulocyte populations. We further explored modulation of the B cell repertoire, its associations with the establishment of a SARS-CoV-2-specific humoral response, and activation of T cells relative to disease severity. Together our results reveal a potential platform for assessing disease trajectory and identify distinct immune perturbation patterns in severe COVID-19 that merit consideration for therapeutic immunomodulation strategies to ameliorate disease severity and organ failure.

## RESULTS

### Demographics and clinical characteristics of moderate and severe COVID-19+ individuals

We recruited 35 inpatients with active COVID-19, seven of whom had moderate disease and 28 with severe disease, seven recovered COVID-19+ donors, and 12 HD. We defined severe disease as requiring oxygen at a flow rate higher than 6 L per minute or by an advanced oxygen delivery device including invasive mechanical ventilation, non-invasive ventilation, or high flow nasal cannula since greater than 6 L is considered high flow oxygen (*16*). All recovered donors reported mild disease and did not receive inpatient care or COVID-19 directed therapy during the course of their illness. For inpatients, median follow up after enrollment was 27 days (range 20 – 43) since blood draw. General demographics and clinical characteristics are shown in Table 1 and Fig. S1A-C. The median ages in the moderate and severe COVID-19+ groups were 59 and 68 years old, respectively, concordant with previous reports (*5*), and were not significantly different (p=0.51). Both the HD and recovered groups were significantly younger than individuals with severe COVID-19+ (p<0.001 in both cases). In line with a recent publication (*6*), the majority of the individuals in the severe and recovered groups were male (67.9% and 71.4%, respectively), while approximately 29% were male in the moderate disease group. The median number of days since onset of symptoms to disease progression in donors with severe COVID-19 was nine, similar to previous publications (*3*, *7*). Individuals with moderate disease also reported a median of nine days since onset of symptoms. In accordance with a recent report (*17*), individuals with COVID-19 had high incidence of underlying pulmonary disease (11/35 including moderate and severe, 31.4%; Fig. S1D) and were current or former smokers (13/35 including moderate and severe, 42.7%, higher in individuals who developed severe disease).

**Table 1:** Demographics and clinical characteristics. Data are shown as number and percentage, n (%). Age is reported in median years (min-max). Days since onset of symptoms is reported as median (min-max). Not all data were collected for HD and recovered individuals. ARDS, acute respiratory distress syndrome; HFNC - NIV, high flow nasal cannula - non-invasive; ECMO, extracorporeal membrane oxygenation.

**Characteristic**	**HD**	**Recovered**	**Moderate**	**Severe^a^**
**n**	12	7	7	28
**Age**	36 (24-61)	30 (20-49)	59 (29-64)	68 (38-81)
**Male**	6 (46.1)	5 (71.4)	2 (28.6)	19 (67.9)
**Race**				
Black or African American	-	-	5 (71.4)	16 (57.1)
Asian or Asian American	-	-	0	2 (7.1)
White or Caucasian	-	-	2 (14.3)	11 (39.2)
**Past smoking history**	-	-	2 (14.3)	13 (46.4)
**Comorbidity**				
Obesity	-	-	3 (42.9)	8 (28.6)
Hypertension	-	-	5 (71.4)	21 (75)
Diabetes	-	-	1 (14.3)	7 (25)
Thromboembolic complications			1 (14.3)	7 (25)
Coronary artery disease/myocardial infarction	-	-	0	3 (10.7)
Underlying lung disease^b^	-	-	4 (57.1)	7 (25)
Renal insufficiency/chronic kidney disease	-	-	2 (14.2)	20 (71.4)
Hyperlipidemia	-	-	2 (71.4)	14 (50)
**Treatment**				
Hydroxychloroquine	-	-	4 (57.1)	25 (89.3)
Remdesivir^c^	-	-	1 (14.2)	12 (42.9)
**Days since onset of symptoms**^d^	-	27 (17-32)	9 (1-16)	9 (1-25)
**Oxygen therapy/ARDS**				
Nasal cannula (oxygen < 6L)	-	-	3 (42.9)	0
HFNC / NIV	-	-	-	4 (4.3)
Ventilator non-ARDS	-	-	-	2 (67.1)
Mild ARDS	-	-	-	3 (10.7)
Moderate ARDS	-	-	-	9 (32.1)
Severe ARDS	-	-	-	10 (35.7)
ECMO	-	-	-	1 (3.6)
**Mortality**	0	0	0	7 (25)

^a^Two severe COVID-19+ individuals were excluded from immunophenotyping and antibody quantification as they displayed clear outlier phenotype due to Rituxan treatment for lymphoma, and acute lymphocytic leukemia, respectively. ^b^Underlying lung disease includes asthma, chronic obstructive pulmonary disease and interstitial lung disease. ^c^Donors enrolled in a clinical trial to test remdesivir versus placebo. Remdesivir was administered after blood collection. ^d^Days since onset of symptoms accounted from the time of blood collection.

Hypertension and hyperlipidemia were the most frequent comorbidities in moderate and severe COVID-19. The majority of individuals with severe COVID-19 presented with moderate and severe ARDS (*18*), and hospital mortality was 25% within this group (Table 1, Fig. S1E). An APACHE III score (*19*) was defined for donors with moderate and severe disease. The APACHE III score was higher in severe vs. moderate disease (p=0.0008) and was directly correlated with age (Fig. S1F-G). In our cohort, high scores were driven primarily by organ failure as clinically determined. Thromboembolic complications, metabolic, vascular and pulmonary disease were also observed more frequently among those with severe disease (Table 1, Fig. S1D). As part of clinical care, D-dimer, procalcitonin, ferritin, lactate dehydrogenase, and C-reactive protein levels were measured in moderate and severe COVID-19+ individuals (Fig. S1H-I). Median levels of D-dimer at the time of blood draw were 3.99 μg/ml in severe, and 0.62 μg/ml in moderate COVID-19 donors (p=0.0022). We found higher levels of ferritin in the severe group compared to the moderate group (p=0.007). Consistent with previous findings (*8*), median procalcitonin values were relatively low, though higher in severe donors than in those with moderate disease (p=0.0014). Levels of lactate dehydrogenase and human serum C-reactive protein (hsCRP) were similar across groups. Bacterial co-infection was present in nine individuals with severe COVID-19, and in only one moderate donor. An extended list of clinical information of the analyzed individuals is shown in Table S1 and summarized in Fig. S1A.

### Immune perturbation in severe COVID-19

**Fig. 1:**
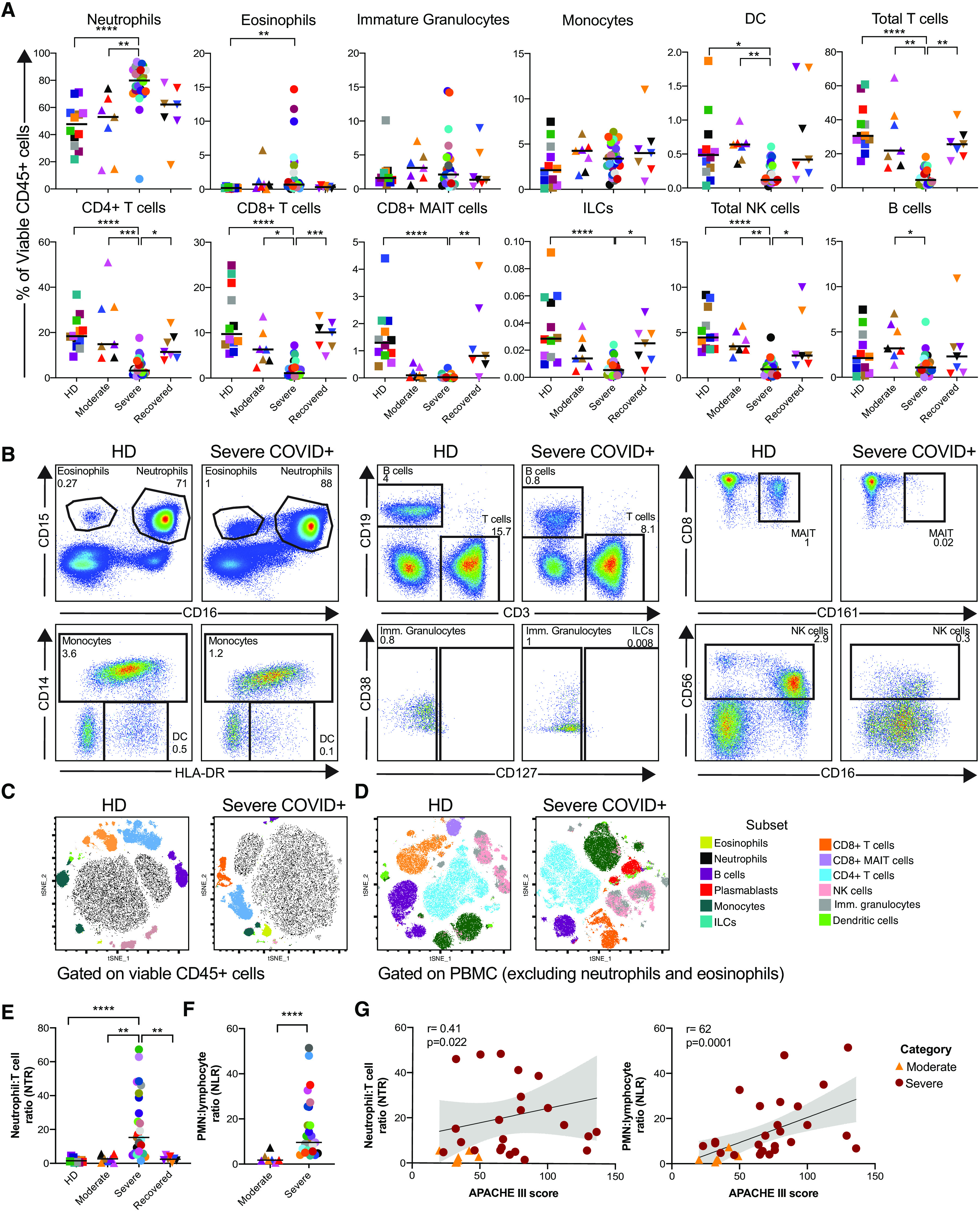
Atlas of immune perturbation in severe COVID-19. Multiparametric flow cytometry analyses on fresh whole blood after red blood cell lysis characterizing immune cells subsets in healthy donors (HD, n= 12), and moderate (n=7), severe (n=27), and recovered (n=7) COVID-19+ individuals. **A)** Subset frequencies were calculated within the total viable leukocyte CD45+ population. **B)** Dot plots for each immune cell subset in a representative HD and severe COVID-19+ individual. Gates within each plot indicate cell subset and corresponding frequency within viable CD45+ cells. Example of parent gates are shown; frequencies were calculated using the specific gating strategies shown in Fig. S2. **C)** Representative examples of the peripheral blood immunologic atlas of a HD and dysregulation within a severe COVID-19+ individual. T-distributed stochastic neighbor embedding (t-SNE) analysis of cell subsets gated on total viable CD45+ cells or **D)** PBMC (viable CD45+ cells excluding neutrophils and eosinophils) on a HD and a severe COVID-19+ individual. **E)** NTR calculated using flow cytometry measurements within viable CD45+ cells. **F)** NLR calculated using CBC counts (Fig. S1I-J). **G)** Spearman correlations of APACHE III score and NTR or NLR in moderate and severe COVID-19+ donors. Differences between groups were calculated using Kruskal-Wallis test with Dunn’s multiple comparison post-test. **** p<0.0001, ***p<0.001, **p<0.01, *p<0.05.

To assess the general landscape of immune responses and their perturbation during severe COVID-19, we performed extensive immunophenotyping to characterize the frequencies of circulating immune subsets in moderate, severe and recovered COVID-19+ individuals compared to HD (Fig. 1, Fig. S2). As previously reported (*7*), the numbers of white blood cells (WBC) and polymorphonuclear leukocytes (PMN) were elevated above normal in all COVID-19+ individuals, and were significantly higher in donors with severe over moderate disease, as measured clinically by complete blood counts (CBC; Fig. S1I-J). We observed an expansion in the proportion of both neutrophil and eosinophil populations in severe COVID-19+ donors compared to HD (p<0.0001 for neutrophil, and p=0.0015 for eosinophil frequencies; Fig. 1A-C). The neutrophil frequency also differed significantly between moderate vs. severe COVID-19 disease (p=0.0046), but did not show increased activation or cycling (Fig. S3A). Furthermore, we saw decreased expression of CD15 in neutrophils between HD and severe COVID-19+ individuals (p=0.0095), but not in eosinophils (Fig. S3B). We did not find significant differences in the immature granulocyte frequencies between HD and COVID-19+ individuals. However, the proportion of immature granulocytes in moderate and severe COVID-19+ donors correlated inversely with the time since onset of symptoms (Fig. S3C). The monocyte blood counts were also higher than normal values in COVID-19+ donors (Fig. S1I-J). However, in contrast to previous work (*20*), the total proportion of monocytes (CD14+ HLA-DR+), as well as monocyte subsets (defined by CD14 and CD16), was similar across groups (Fig. 1A and S3D-E). Donors with severe COVID-19 had lower frequencies of dendritic cells (DC) compared to moderate disease (p=0.003) and HD (p=0.0374; Fig. 1A), but not with recovered individuals. This decrease was observed in both conventional (CD11c+ CD123lo/-) and plasmacytoid (CD11c- CD123+) DC subsets (Fig. S3F).

Consistent with previous reports (*4*, *5*, *21*, *22*), we observed a relative decrease in the percentages of all lymphocyte subsets (Fig. 1A, B, D and Fig. S1I-J for lymphocyte absolute count). Severe COVID-19+ individuals had significantly lower proportions of T cells (p<0.0001), as well as total CD4+ and CD8+ T cells. We also observed lower frequencies of CD8+ MAIT cells, innate lymphoid cells and natural killer (NK) cells than HD (p<0.0001 for all cases). We did not find significant differences in the frequencies of these cell subsets between HDs and moderate or recovered COVID-19+ individuals. Within the NK cell lineage, we observed a drastic decrease in the frequencies of both CD56brightCD16- and CD56dimCD16+ NK cells in severe COVID-19 vs. HD (Fig. S3G). In the recovered group, the proportion of T cells, CD8+ MAIT cells, ILCs and NK cells were comparable to HDs. Lymphocyte frequencies were inversely correlated with APACHE III score on COVID-19+ donors (Fig. S1K). We initially defined CD8+ MAIT cells based on their bright expression of CD161 (Fig. S2B). To ensure that these CD161++ CD8+ T cells were indeed MAIT cells, we quantified their expression of TCR Vα7.2 and found that ~80% of CD161++ CD8+ expressed this marker in both HD and COVID-19+ donors (Fig. S4A-B) allowing us to consider them primarily MAIT cells. The proportions of circulatory follicular helper CD4+ T cells (cTfh) and regulatory CD4+ T cells were similar across all groups (Fig. S4C-D). Although we did not observe differences in CD4+ and CD8+ memory T cell subsets between groups, we did find a negative correlation with the frequency of central memory T cells (T_CM_) and days since the onset of symptoms (Fig. S4E).

The neutrophil-to-lymphocyte ratio (NLR) has been proposed to be an independent risk factor for severe disease (*23*); therefore we examined the neutrophil:T cell ratio (NTR) based on their frequencies within viable CD45+ cells, as well as the ratio of PMN:lymphocytes as measured clinically (NLR, Fig. 1E-F). Individuals with severe COVID-19 had a median NTR of 15, while all other studied groups had ratios of less than 2.6 (Fig. 1F). Of note, NLR and NTR were strongly correlated (r=0.64, p<0.0001), thus validating our finding with two independent measurements. Both NLR and NTR positively correlated with APACHE III score in COVID-19+ donors (Fig. 1G) independently of age, further emphasizing a potential role of this ratio as a biomarker of disease severity. Finally, we observed a significant association between NLR and severe COVID-19 that was independent of age or vascular comorbidity (odds ratio 1.66, 95% confidence interval 1.02-1.70, p=0.042). The NTR was also associated with disease severity (odds ratio 1.94, 95% confidence interval 1.04-3.62, p=0.036) although this association was attenuated by adjustment for age and vascular disease (odds ratio 2.12, 95% confidence interval 0.94–4.78, p=0.07). Altogether, these data reveal multiple immunophenotypic abnormalities in severe COVID-19 that are not found in donors with moderate or recovered disease, and confirm the prognostic utility of NLR/NTR.

### Elevated frequency of plasmablasts, changes in B cell subsets and humoral responses

**Fig. 2:**
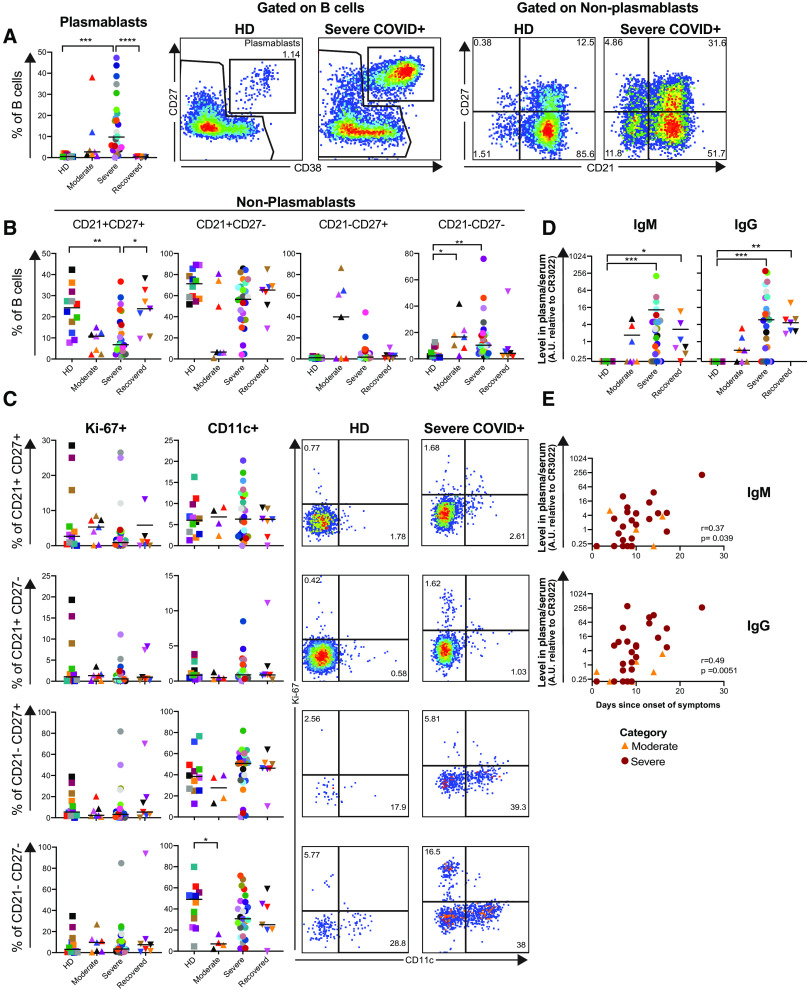
Elevated frequency of plasmablasts, changes in B cell subsets and SARS-CoV-2-specific antibody production in COVID-19+ individuals. Multiparametric flow cytometry analyses on fresh whole blood after red blood cell lysis characterizing plasmablast and B cell subset frequencies from HD (n= 12), and moderate (n=7), severe (n=27), and recovered (n=7) COVID-19+ individuals. **A), B)** Distribution and representative plots of B cell plasmablasts (defined as CD27+ CD38+ B cells) and non-plasmablast subsets defined by CD21 and CD27 expression in HD (n= 12), and moderate (n=7), severe (n=27), and recovered (n=7) COVID-19+ individuals. Numbers inside the plots indicate the subset proportion of the corresponding parent population (within total B cells for plasmablasts, within non-plasmablasts for CD21/CD27 subsets). **C)** Frequencies of K_i_-67 and CD11c in non-plasmablast B cell subsets defined in a). Analyses of CD11c are shown for 4/7 individuals with moderate COVID-19. Plots from a representative HD and severe COVID-19+ individual shown. Numbers in each plot indicate the frequency within the parent gate. **D)** Levels of SARS-CoV-2 spike RBD-specific IgM and IgG antibodies in serum or plasma of HD (n=12), moderate (n=7), severe (n=27), and recovered (n=7) COVID-19+ individuals. Antibody measurements were performed by ELISA using plates coated with the receptor binding domain (RBD) from the SARS-CoV-2 spike protein. Serum and plasma samples were heat-inactivated at 56°C for 1 hour prior to testing in ELISA to inactivate virus. Antibody levels were measured as IgG and IgM arbitrary units (A.U.) based on O.D. values relative to the CR3022 monoclonal antibody (recombinant human anti-SARS-CoV-2, specifically binds to spike protein RBD). **E)** Spearman correlations of plasma/serum levels of SARS-CoV-2 RBD-specific IgM (top) and IgG (bottom) and days since onset of symptoms on moderate and severe COVID-19+ individuals. Differences between groups were calculated using Kruskal-Wallis test with Dunn’s multiple comparison post-test. **** p<0.0001, ***p<0.001, **p<0.01, *p<0.05.

Although we observed only marginal differences in the proportions of total B cells between the studied groups (Fig. 1), B cell plasmablasts were significantly expanded in severe COVID-19+ donors compared to HD (p<0.0001; Fig. 1D, Fig. 2A;). These cells characteristically displayed high levels of K_i_-67 and low levels of CXCR5 expression (Fig. S5A). Similar to observations in the immune atlas of recovered COVID-19+ donors (*11*), expanded plasmablasts were not found in this group (p<0.0001 in recovered vs. severe donors). The frequency of plasmablasts in individuals with severe COVID-19 did not correlate with age, days since onset of symptoms or the presence of comorbidities, APACHE III score (Fig. S1L) nor frequency of CD4+ cTfh cells (Fig. S5B).

In the non-plasmablast B cell population, we observed a decrease in the percentage of CD21+CD27+ in moderate and severe groups compared to HD. These proportions were highly significant by nonparametric test of trend (p=0.0008), but only the severe COVID-19 group reached statistical significance vs. HD (p=0.0061, Fig. 2B). Moreover, the overall expression of CD21 in non-plasmablasts (measured by median fluorescence intensity, MFI) was also decreased in both the moderate and severe COVID-19+ groups (Fig. S5C). Recovered COVID-19 donors had similar levels of CD21+CD27+ non-plasmablasts as the HD group. Of note, the frequency of CD21+CD27+ non-plasmablasts was directly correlated with donor age among moderate and severe COVID-19 (Fig. S5D). In contrast, we observed a significant increase in the proportion of CD21-CD27- non-plasmablasts in moderate and severe COVID-19+ individuals compared to HD (p=0.0182 and p=0.004, respectively). We next assessed the expression of K_i_-67 and CD11c, to determine if any of these subsets were a potential source for the expanded plasmablast population (*24*) (Fig. 2C). We did not observe a larger proportion of cycling K_i_-67+ CD21-CD27- B cells in moderate or severe COVID-19+ individuals when compared with HD. We also found a reduction in the frequency of CD11c+ cells within CD21-CD27- B cells in donors with moderate COVID-19 compared to HD that was specific to this group (p=0.0162).

Previous work has suggested that the SARS-CoV-2 IgG levels could be associated with disease severity (*15*, *25*). The levels of total IgG in plasma and serum were equivalent across the groups (Fig. S5E), despite the heightened plasmablast response in severe COVID-19. The levels of SARS-CoV-2 spike receptor binding domain (RBD)-specific IgM and IgG were significantly higher in the severe and recovered COVID-19+ individuals (Fig. 2D). While the frequency of plasmablasts did not correlate with the levels of spike RBD-specific IgM or IgG, there was a positive association between the levels of spike RBD-specific IgM and IgG and time since onset of symptoms (Fig. 2E) in the moderate and severe groups. Together these data indicate an exacerbated plasmablast response in severe COVID-19, as well as the development of a strong SARS-CoV-2-specific humoral response.

### Profound oligoclonal expansion of B cells in severe COVID-19

Having observed the expansion of plasmablasts in severe COVID-19+ donors, we sought to determine whether this expansion in severe COVID-19 resulted from non-specific stimulation. Therefore, we sequenced the antibody repertoire (variable (VH) gene sequence and entire third complementarity determining region (CDR3) from genomic DNA) of randomly selected HD (n=3), moderate COVID-19+ (n=3) and severe COVID-19+ (n=7) individuals. After quality control and filtering, the processed antibody heavy chain rearrangements were grouped together into a data set comprising 76 sequencing libraries and 109,590 clones across all 13 individuals (Table S2 and GenBank/SRA PRJNA630455).

**Fig. 3:**
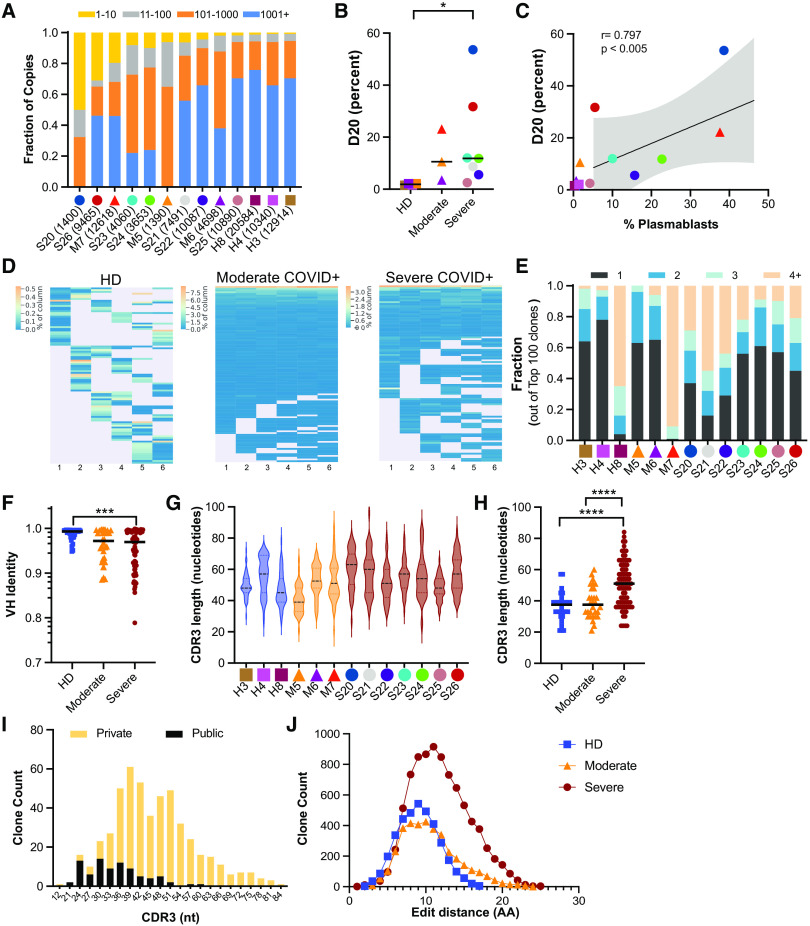
**Abundant antibody heavy chain sequences from severe COVID-19+ individuals have long, diverse CDR3 sequences and higher levels of somatic hypermutation. A)** Clone size distribution by sequence copies. For each donor, the fraction of total sequence copies occupied by the top ten clones (yellow), clones 11-100 (grey), 101-1000 (orange) and over 1000 (blue) are shown. Total donor level clone counts are given in parentheses. **B)** Percentage of sequence copies occupied by the top twenty ranked clones (D20) shown for HD (n=3) and COVID-19+ individuals with moderate (n=3) and severe disease (n=7). **C)** Spearman correlation between the D20 value and the percentage of plasmablasts within the total B cell population. **D)** Examples of the overlap of top 100 copy rearrangements that overlap in at least two sequencing libraries in HD (H4), a moderate COVID-19+ (M7) and a severe COVID-19+ individual (S21). Each horizontal string is a rearrangement and each column is an independently amplified sequencing library (see Materials and Methods). Lines are heat mapped by the copy number fraction for a given replicate library. **E)** Clone size estimation based on sampling (presence/absence in sequence libraries). Shown are the fractions of the top 100 clones that are found in 4 or more sequencing libraries, 3 libraries, 2 libraries and 1 library. All donors had six sequencing libraries, except for M5 (four libraries). **F)** Fractional identity to the nearest germline VH gene sequence (1.0 = unmutated) in the top 10 copy number clones of each donor. Each symbol is a clone. **G)** CDR3 length distributions of the top 50 productive rearrangements in each donor. **H)** CDR3 lengths of the top 10 copy number clones (symbols), stratified by condition. **I)** CDR3 length distribution of top 50 clones in COVID-19+ donors based on whether they are found in the Adaptive database (public) or not (private). **J)** Distribution of CDR3 amino acid (AA) edit distances of the top 50 copy clones (productive) per donor. Clone pair counts for each edit distance are averaged across all the donors in each disease category. Differences between groups were calculated using Mann-Whitney rank-sum test. **** p<0.0001, ***p<0.001, *p<0.05.

To evaluate the clonal landscape, we ranked the proportion of clones within the top ten (1-10), next 90 (11-100), next 900 (100-1,000), and most diverse clones with ranks above 1,000 (1,000+) (Fig. 3A). Donors with severe COVID-19 had an unusually high proportion of large clones comprising the majority of their circulating antibody repertoire, with the fraction occupied by the top 20 ranked clones (D20 measure) the highest compared to the HD and moderate SARS-CoV-2 infected patients (Fig. 3B, Fig. S6) The D20 rank measure in moderate and severe disease also correlated positively with the plasmablast fraction (Fig. 3C). In many severe COVID-19+ individuals we observed very large top copy clones, exceeding the diagnostic thresholds for clinically significant monoclonal B cell lymphocytosis (*26*). These large clones were readily sampled across multiple independently amplified and sequenced libraries (Fig. 3D). Donors M7 and S21 had 91 and 55 clones present in 4 or more sequencing libraries, respectively, in contrast to H4, who had 3 clones in 4 or more libraries (Fig. 3E). Only one HD (H8), an older individual, had large and readily resampled clones, likely reflecting age-dependent narrowing and expansion of the memory B cell repertoire (*27*).

To determine if the antibody heavy chain sequences harbored any evidence of extensive somatic hypermutation (SHM), selective VH gene usage, or defining CDR3 characteristics, we assessed these properties in the top copy clonotypes of each individual. A subset of individuals with severe COVID-19 exhibited higher levels of SHM (Fig. 3F), but other top copy clones in severe COVID-19, moderate COVID-19 and HD were unmutated. To determine if antibodies from COVID-19+ individuals exhibited convergent sequence features, we analyzed VH gene usage in all clones of each donor (Fig. S7A). As this analysis did not reveal any consistent increased usage of a specific VH gene in the moderate or severe COVID-19+ individuals compared to controls, we reanalyzed the data focusing on the top 200 most frequent clones in each individual (Fig. S7B). Focusing on these, VH genes from different families were used more often in severe COVID-19+ donors compared to HD, including VH6-1 (7-fold), VH3-48 and VH3-15 (~6-fold) and several others (Fig. S7C). We also looked for skewing in VH family usage, which revealed a modest relative increase in the proportion of VH3 family members among COVID-19+ individuals compared to HD (Fig. S7D). However, there was considerable inter-individual variation in the usage of VH3 vs. other family members, with some individuals (such as S25) exhibiting substantial skewing toward particular VH families.

Given the absence of obvious or uniform VH restriction among COVID-19+ individuals, we next analyzed the CDR3 sequences for shared characteristics in the COVID-19+ donors. In individuals with severe disease, CDR3 sequences exhibited greater variation in length (Fig. 3G), and were significantly longer among the top copy sequences (Fig. 3H). To determine if the antibody heavy chain sequences from COVID-19+ individuals are generated commonly or infrequently, we searched the Adaptive Biotechnologies public database, which consists of 37 million antibody heavy chain sequences (*28*), revealing 3995 matches to the CDR3 amino acid sequences in our dataset. Among the 50 most frequent clones in the COVID-19+ individuals, the CDR3 lengths of the matching or “public” clones were shorter than the CDR3 lengths of the non-shared or “private” clones (Fig. 3I), indicating that the top copy clones in COVID-19 with long CDR3 sequences are mostly private. Finally, to determine if there were any collections of clones that harbored similar CDR3 amino acid sequences, we computed the edit distances of all of the amino acid sequences in the top 50 clones of each of the individuals. If there were sequence convergence, we would have expected to find clusters of sequences separated by three or fewer amino acids. We found no evidence of co-clustering of CDR3 sequences; rather, over 99% of the edit distances for the severe COVID-19+ individuals’ top copy clone pairs were more than three amino acids apart (Fig. 3J). Consistent with this finding, alignment of top copy clone CDR3 amino acid sequences from severe COVID-19+ individuals revealed highly variable amino acid sequences (Fig. S7E). Taken together, these data show that severe COVID-19 is associated with large, oligoclonal B cell expansions with antibodies enriched for long and divergent CDR3 sequences.

### Innate immune dysregulation in severe COVID-19

**Fig. 4:**
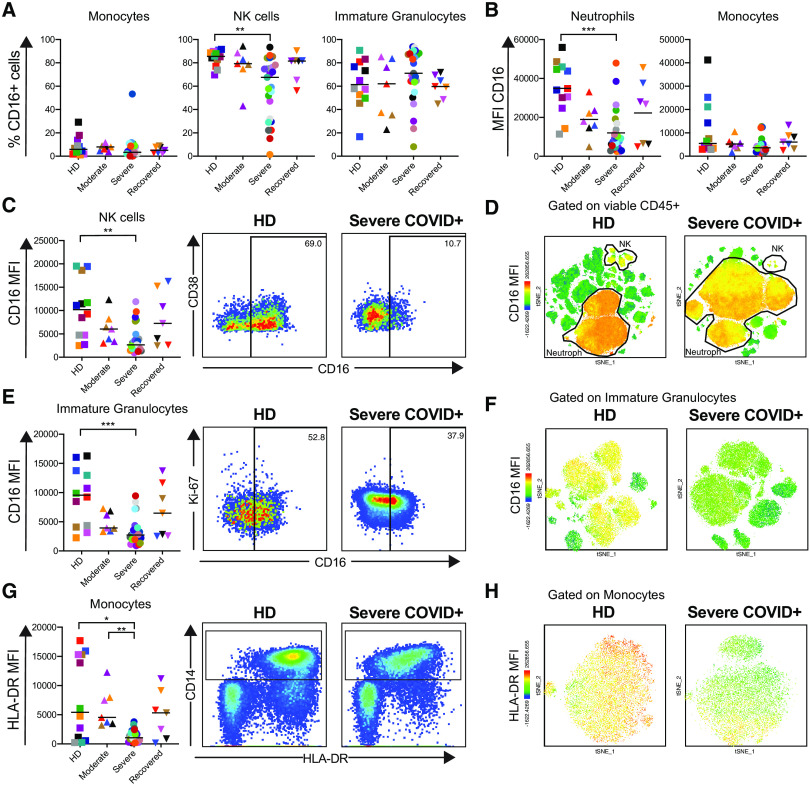
Innate immune dysregulation in severe COVID-19. Multiparametric flow cytometry analyses of fresh whole blood after red blood cell lysis characterizing the expression of CD16 and HLA-DR on innate immune cells from HD (n= 12), moderate (n=7), severe (n=27), and recovered (n=7) COVID-19+ individuals. **A)** Proportion of CD16+ cells in monocyte, NK cell and immature granulocyte subsets. **B), C), E)** Median fluorescence intensity (MFI) of CD16 on neutrophil, monocyte, NK cell and immature granulocyte subsets. MFI was calculated within CD16+ cells. Representative dot plots showing CD16 expression in NK cells and immature granulocytes of a HD and a severe COVID-19 individual shown in **C)** and **E)**. The numbers inside the plots indicate the percentage of CD16+ cells in the corresponding parent population. **D), F)** t-SNE analyses of CD16 expression (MFI) in viable CD45+ cells or immature granulocytes, respectively, on a representative HD and a severe COVID-19+ individual. **G)** MFI of HLA-DR on monocytes; dot plots of a representative HD and a severe COVID-19+ individual shown, with monocyte gate outlined. **H)** t-SNE analyses of monocyte HLA-DR expression (MFI) on a representative HD and a severe COVID-19+ individual. Differences between groups were calculated using Kruskal-Wallis test with Dunn’s multiple comparison post-test. ***p<0.001, **p<0.01, *p<0.05.

Acknowledging the characteristic differences in innate cell subset frequencies in individuals with severe COVID-19 (Fig. 2), we further assessed the phenotype of innate immune cells. CD161 has been reported to be a marker of inflammatory monocytes and NK cells (*29*, *30*). Despite having observed a decreased frequency of CD8+ MAIT cells (Fig. 2A, D), the frequencies of CD161+ monocytes and CD38+CD161+ NK cells were similar across study groups (Fig. S3H). We next assessed the frequency and expression of CD16 by neutrophils, monocytes, NK cells and immature granulocytes. While the proportions of CD16+ monocytes and immature granulocytes were consistent between groups, severe COVID-19+ individuals had significantly lower circulating CD16+ NK cells in compared with HDs (p=0.0023; Fig. 4A; also observed when analyzing NK cell subsets in Fig. S3G). Furthermore, CD16 expression was significantly lower in neutrophils, NK cells, and immature granulocytes in severe COVID-19 compared to HD (p<0.002 for all cases, Fig. 4B-F). Down-regulation of CD16 in NK cells has been associated with IgG-mediated immune complexes in the context of vaccination (*31*). We did not, however, find significant associations between the frequency or expression of CD16 and IgG levels (Fig. S3I). To determine whether CD16 was down-regulated or CD16+ NK cells were lost, we further examined expression of other canonical NK cells markers within the total CD3- CD56+ population. Irrespective of CD16, the majority of CD56+ NK cells expressed NKG2A/2C, NKp30 and NKp46 (Fig. S3J), suggesting down-regulation of CD16 rather than specific depletion of CD56low CD16+ NK cells. To assess the possibility of CD16 internalization, we measured the CD16 expression on the cell surface and after cell membrane permeabilization (Fig. S8) and found no significant difference between stains. This suggests that loss of cell surface CD16 expression during COVID-19 is not only due to receptor internalization, and that other mechanisms may be responsible, such as transcriptional down-regulation or shedding. Indeed, Wilk *et al*., recently reported a decrease in CD16 mRNA on NK cells during COVID-19 (*11*).

Although we found a decrease in the frequency of CD16+ monocytes in some severe COVID-19+ individuals, this was not consistent amongst the whole cohort (Fig. S3D-E). Monocyte CD16 expression level tended to decrease with disease severity (p=0.022 by nonparametric test of trend; Fig. 4B). However, monocytes significantly down-regulated HLA-DR expression in severe COVID-19+ donors compared to moderate disease (p=0.0072) and HD (p=0.021; Fig. 4G-H). Similar findings were reported by scRNASeq analysis of severe COVID-19+ individuals (*11*) and donors with severe respiratory failure (*32*). In contrast, CD14 expression in monocytes or HLA-DR in other antigen presenting cells (Fig. S3K-L) was consistent across all studied groups. Altogether, these findings indicate a substantial perturbation of the innate immune system in severe COVID-19. Whether this dysregulation is consequence or contributing factor toward COVID-19 severity remains to be defined.

### Heterogeneous T cell activation in severe COVID-19

**Fig. 5:**
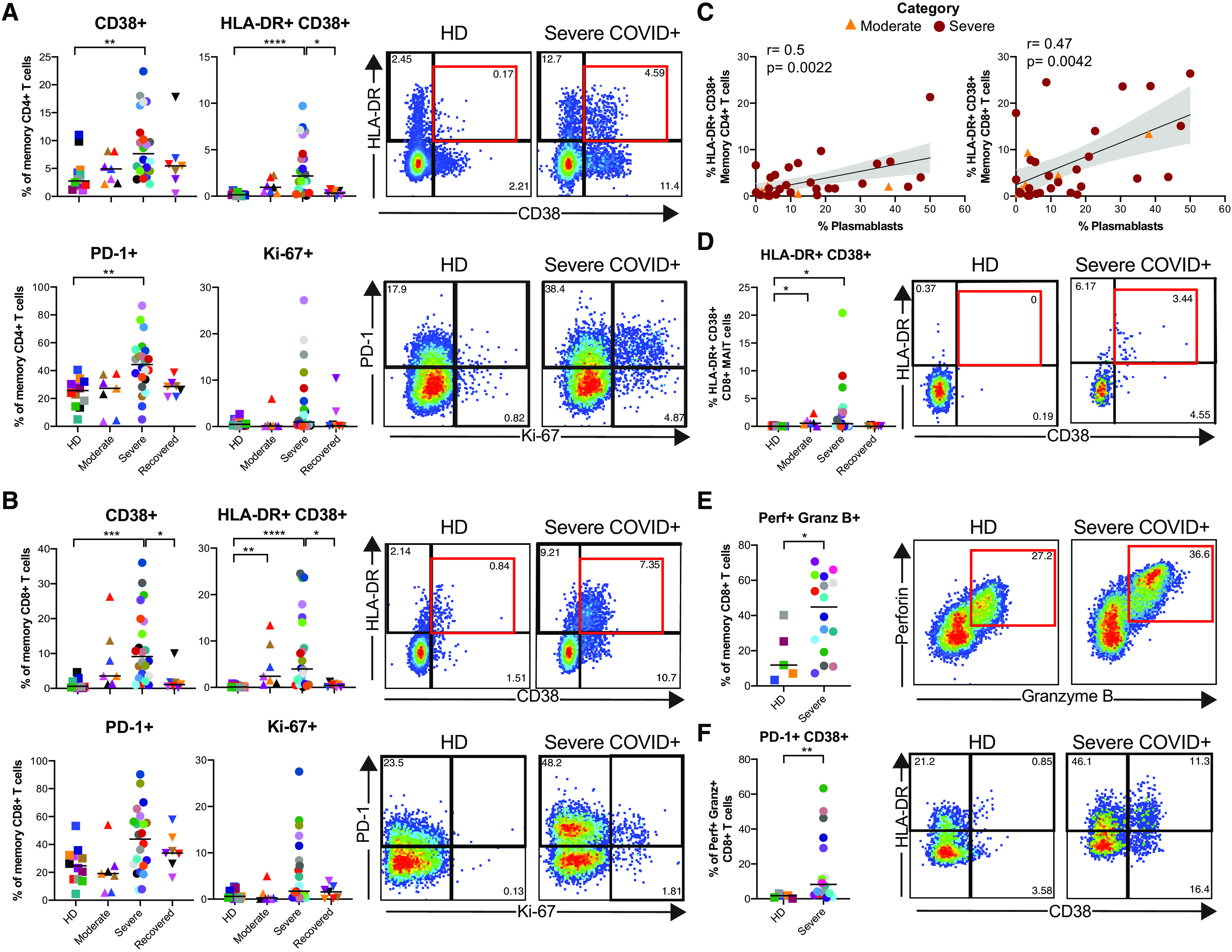
Heterogeneous T cell activation in severe COVID-19. Multiparametric flow cytometry analyses on fresh whole blood after red blood cell lysis characterizing immune cells subsets in HD (n= 12), moderate (n=7), severe (n=27), and recovered (n=7) COVID-19 individuals was performed to assess the percentage of activated memory T cells. Frequencies of CD38+, HLA-DR+CD38+, PD-1+ and Ki67+ in **A)** CD4+, and **B)** CD8+ memory T cells (excluding naïve CCR7+ CD45RA+, detailed gating strategy shown in Fig. S2). **C)** Spearman correlations between the frequencies of HLA-DR+CD38+ CD4+ or CD8+ memory T cells and plasmablasts in donors with moderate (orange triangles) or severe COVID-19 (dark red circles). **D)** Frequencies of HLA-DR+CD38+ CD8+ MAIT cells. **E)** Frequency of cytotoxic memory CD8+ T cells. Multiparametric flow cytometry analyses were performed on freshly isolated PBMC from HD (n=5) and severe (n=16) COVID-19+ individuals to quantify the frequency and phenotype of cytotoxic (as defined by perforin and granzyme B expression). **F)** CD8+ T cells, and proportion of cytotoxic CD8+ T cells expressing PD-1 and CD38. Plots for a representative HD and a severe COVID-19+ individual are shown. Numbers inside the plots indicate the frequency within the corresponding parent population. Differences between groups were calculated using Kruskal-Wallis test with Dunn’s multiple comparison post-test and Mann-Whitney rank-sum test. **** p<0.0001, ***p<0.001, **p<0.01, *p<0.05.

T cell activation has been reported in acute respiratory and non-respiratory viral infections (*33*, *34*). Consistent with recent case reports (*10*), we observed increased activation of both memory CD4+ and CD8+ T cells in severe COVID-19+ individuals compared to other study groups (Fig. 5A and B). However, unlike the plasmablast response, heightened T cell activation was not observed in every severe COVID-19+ individual and instead demonstrated significant heterogeneity. Overall the frequencies of CD38+ and HLA-DR+CD38+ memory CD4+ and CD8+ T cells in severe COVID-19 were elevated compared to HD (p<0.01 for all cases), and frequency of HLA-DR+ CD4+ and CD8+ T cells was also directly associated with APACHE III scores (Fig. S1M). While we did not find statistically higher K_i_-67+ CD4+ or CD8+ T cells in COVID-19 individuals compared to HD, a subset of severe COVID-19+ donors clearly had increased levels of K_i_-67+ CD4+ and CD8+ T cells, reaching as high as ~25% in some individuals. The frequency of PD-1+ memory CD4+ T cells (p=0.0084), but not CD8+ T cells, was also higher in the severe COVID-19+ group compared to the HD group. For all measures, CD4+ and CD8+ T cell activation in recovered donors was equivalent to the HD group. The proportion of PD-1+ memory CD4+ T cells, but not of PD-1+ CD8+ T cells, in moderate or severe COVID-19 correlated with donor age (Fig. S4F). The frequency of PD1+ CD4+ T cells was also correlated with APACHE III (Fig. S1M). In addition, the frequencies of HLA-DR+CD38+ CD4+ and CD8+ T cells correlated with the proportion of plasmablasts in moderate and severe COVID-19+ individuals (Fig. 5C). The heterogeneity of T cell activation and its association with B cell responses and clinical parameters is thoroughly explored in the companion study (*35*). We further examined activation of specific memory CD4+ and CD8+ T cell subsets (Fig. S9), where we found that many of the individual memory subsets expressed higher levels of activation markers selectively in the severe COVID-19 patients. The frequency of activated CD4+ cTfh cells was also increased in a large proportion of donors with severe COVID-19 (Fig. S4G) but did not associate with plasmablast frequency.

Given the reported role of CD8+ MAIT cells in COVID-19 severity (*36*) and the decrease observed in both moderate and severe COVID-19+ individuals (Fig. 1, Fig. S4A-B), we further analyzed CD8+ MAIT cell activation. Similar to total CD4+ and CD8+ T cells, CD8+ MAIT cells in severe COVID-19 displayed significantly heightened activation for many of the measured markers, including HLA-DR (p=0.049 between severe and HD), CD38+ (p=0.048 between severe and HD), and CD69 (p=0.04 between severe and recovered) in severe COVID-19 (Fig. 5D and Fig. S4H), but not for PD-1 or K_i_-67 as a cohort. However, consistent with total memory CD4+ and CD8+ T cells, CD8+ MAIT cells in some severe COVID-19+ individuals displayed greatly elevated expression of the various activation markers measured.

We further quantified the proportion of cytotoxic CD8+ T cells (defined as perforin+ granzyme B+ memory CD8+ T cells, Fig. 5E) in a subset of HD and severe COVID-19+ individuals. Due to limited samples, we did not include the moderate or recovered COVID-19+ groups for this analysis. We found a significantly higher proportion of cytotoxic CD8+ T cells in severe COVID-19 than in HD (p=0.048). The frequencies of T-bet+ cells, as well as the levels of expression of perforin+ and granzyme B+ cells within the cytotoxic memory CD8+ T cell subset were similar between groups (Fig. S4I-J). Cytotoxic CD8+ T cells from severe COVID-19+ donors also had an increased proportion of cells expressing CD38 or co-expressing PD-1 and CD38 compared to HD (p=0.0082; Fig. 5F and Fig. S4K). These data indicate a heightened status of immune activation and frequency of cytotoxic CD8+ T cells during severe COVID-19.

### Distinctive severe COVID-19 immunophenotype

**Fig. 6:**
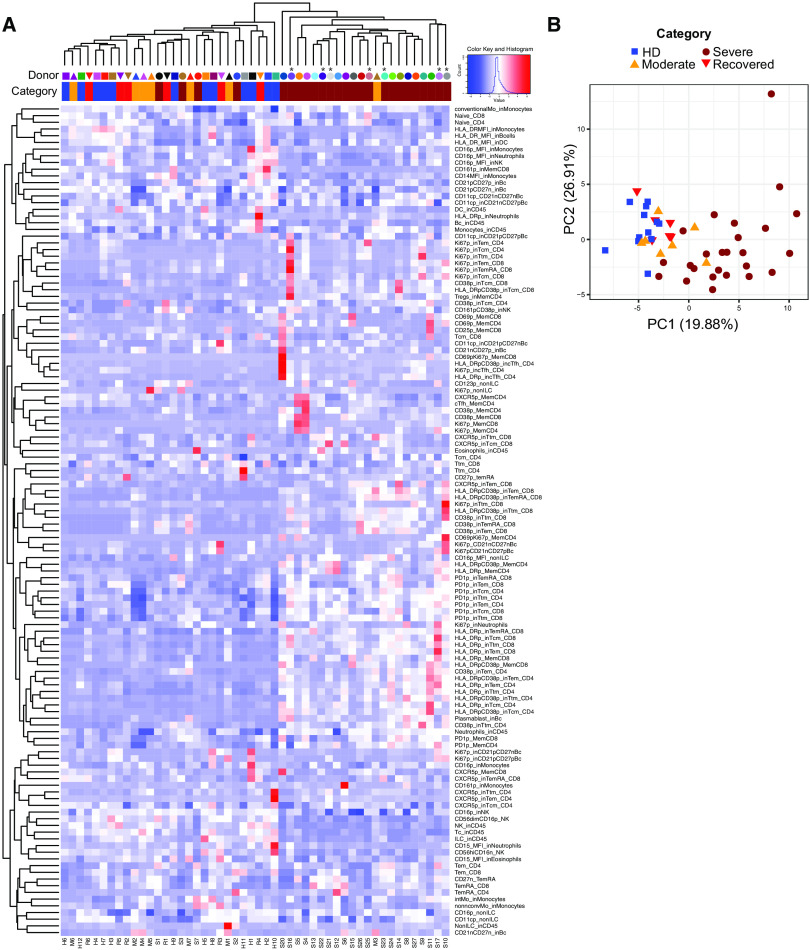
**Unbiased analyses of immunophenotyping reveals selective clustering of severe COVID-19+ individuals. A)** Heatmap of flow cytometric analyses of HD (n= 12), moderate (n=7), severe (n=27), and recovered (n=7) COVID-19+ individuals. Data are shown in z-score scaled values. Shape and color coding correspond to data shown in Figs. 1-6. H, HD; M, moderate COVID-19; S, severe COVID-19; R, recovered COVID-19. Stars above the symbols indicate donors who died during hospitalization. **B)** Principal component analysis generated using all flow cytometric data from **A)**.

Finally, we performed an unbiased analysis to determine if the immune cells in the severe COVID-19+ disease cohort could be differentiated from the HD, moderate, and recovered cohorts. We included all analyzed immune phenotype parameters described thus far. We scaled all flow cytometry generated data using z-score, and performed hierarchical clustering (Fig. 6A). From this analysis, the data from 21/28 of the severe COVID-19+ patients co-localized to a distinct cluster within the hierarchical tree. We further analyzed these data by principal component analysis, where we again found selective clustering of individuals with severe COVID-19 (Fig. 6B). The top parameters driving the clustering of the severe COVID-19 were associated with T cell activation in CD4+ and CD8+ T cell memory subsets, frequency of plasmablasts and frequency of neutrophils (Table S3), also evidenced in the heat map shown in Fig. 6A. Independent analyses of the severe COVID-19+ group did not produce separate sub-clustering, likely due to reduced sample number. Deceased individuals did not cluster separately either. However, it is clear from the heatmap analysis that distinct patterns within the severe COVID-19+ cohort may be present that further subdivide these individuals into different subgroups. Taken as a whole, our analysis reveals a characteristic immune phenotype in severe COVID-19, distinct not only from HD but also from other COVID-19+ individuals with moderate or recovered disease.

## DISCUSSION

Devising therapeutic strategies to treat SARS-CoV-2 infection remains challenging, due to both the complexity of the clinical manifestations and an overall lack of understanding of severe COVID-19 immunopathogenesis. Reports on single individuals, studies with small patient numbers of varying disease stages, or focused analyses on limited immune phenotypes have generated valuable information, but have fallen short of providing a comprehensive immunophenotypic atlas of severe COVID-19. Here, we sought to define immune perturbations of COVID-19 in moderate and severe disease using an unbiased approach, finding profound changes in multiple leukocyte populations selectively in severe disease. Together, these data provide both insights into the immunopathogenesis of severe COVID-19, including pronounced effects on neutrophils, monocytes, NK cells, and B and T lymphocytes.

Retrospective clinical metadata studies (*23*) have identified an elevated NLR in severe COVID-19. We identified a similar association here, with both NLR and NTRs being highly elevated in patients with severe COVID-19 patients. These ratios also correlated directly with the APACHE III score, an independent metric of morbidity used in clinical trials as an assessment of predicted mortality at ICU admission (*19*). In our cohort, donors with higher APACHE III score presented with hematologic or metastatic malignancy, immunocompromised cirrhosis, hepatic failure with encephalopathy, and the high scores were driven primarily by organ failure data. The strong correlation of NLR, validated by clinical data and flow cytometric analysis, with APACHE III suggests that NLR could be used as a biomarker of risk for multi-organ failure or death.

Modulation of innate immune cells in severe COVID-19 manifested in a number of ways, including broad changes in the frequency and phenotype of circulating neutrophils, monocytes and NK cells typified by down-regulation of CD15 and CD16 on neutrophils, and CD16 on NK cells, immature granulocytes and monocytes. It is unclear whether CD15 and CD16 down-regulation in these cell types marks an activated or refractory state. CD16 expression has been suggested to have diagnostic value in neutrophil left shift, inflammatory and infectious diseases (*37*). Furthermore, neutrophilia observed in COVID-19 may be a source of excess neutrophil extracellular traps (NETs), markers for which are elevated in the sera of COVID-19+ individuals (*38*). The frequency of neutrophils was one of the main drivers of independent clustering of individuals with severe COVID-19 in our unbiased analysis. How the phenotypic changes observed here impact the neutrophil ability to produce NETs and the association with disease severity remain to be determined. CD16 down-regulation has been associated with NK cell maturation and development (*39*), as well as with activation and target cell engagement, resulting in antibody dependent cell cytotoxicity (ADCC) and TNF-alpha secretion. Down-regulation of CD16 after interaction with IgG-immune complexes also may prevent excessive immune responses after influenza vaccination (*31*, *40*). As such, the marked CD16 down-regulation we observed on NK cells in severe COVID-19 patients suggests substantial mobilization of these cells. The implications of the observed changes in the expression of CD15 in neutrophils, as well as CD16 across subsets during severe COVID-19 and their potential role as indicators of redistribution to the lungs, link with function and response, as well as diagnostic and prognostic significance (*41*, *42*), requires additional exploration.

One of our most striking findings was a profound expansion of plasmablasts during severe COVID-19, in some patients rivaling or exceeding that observed in acute hantavirus, dengue and Ebola infections or chronic inflammatory conditions such as systemic lupus erythematosus (*33*, *43*, *44*). One recent study suggested that COVID-19+ individuals in critical condition show extrafollicular B cell activation (*45*). The increase in plasmablast frequency we observed directly correlated with an oligoclonal expansion of antibody clones within the overall B cell repertoire, suggesting that many of these large clonal expansions reside within the plasmablast pool. Remarkably, in some severe COVID-19+ individuals a single clone could account numerically for the entire plasmablast population. Although oligoclonality is known to increase in older individuals, the levels of oligoclonality we observed exceeded that in our older control, and those commonly used in a clinical setting to define monoclonal B cell lymphocytosis (*46*–*48*). Furthermore, the heavy chain CDR3 length is not substantially increased in the elderly (*47*, *48*) unlike the hospitalized patients with COVID-19 that we studied. The antibody sequences of the largest B cell clones in the severe COVID-19+ individuals were surprisingly variable in terms of SHM levels and VH gene usage. In line with a recent report (*49*), we did not observe clear sequence convergence of VH genes amongst all the severe COVID-19+ individuals, but VH3 family members were enriched in some. Variable VH gene usage and varying levels of SHM could indicate a polyclonal response that is arising via an extrafollicular pathway in COVID-19, consistent with recently reported findings of Sanz and colleagues (*45*). Extrafollicular B cell activation has also been observed in other inflammatory conditions including systemic lupus erythematosus and in the setting of infection, and may result in low-affinity or multireactive antibodies (*50*, *51*). One consistent feature of many of the large clones was the presence of elongated CDR3 sequences compared to clones in donors with moderate COVID-19 and HD. Long CDR3 sequences are infrequent in the primary repertoire because they are difficult to generate and they are often multireactive and counter-selected during B cell development (*52*). Individuals with reduced diversity in their primary repertoire (including the elderly and immunocompromised) might be at increased risk for severe disease if there is a bottleneck to their production. It is also possible that antibodies with long CDR3 sequences could be harmful and are either multireactive or arise as a result of bystander activation in the setting of inflammation (*53*–*56*). Multireactive antibodies may, in turn, form damaging immune complexes that promote the release of additional autoantigens from injured or dying cells, amplifying tissue injury. CDR3 sequences from individuals with severe COVID-19 had higher edit distances than individuals with mild disease or HD. While their size, somatic mutation status and association with the plasmablast fraction are suggestive of active participation in the immune response to SARS-CoV-2, it is unknown if these clones can recognize the virus, confer protection, or contribute to immunopathology.

Individuals infected with coronavirus mount neutralizing antibody responses, and this has been recently confirmed for SARS-CoV-2 (*57*–*60*). Levels of SARS-CoV-2 spike RBD-specific antibodies are directly associated with neutralizing antibodies from day 9 onwards (*61*). Furthermore, a recent case report showed that extensive maturation is not necessary for neutralization (*60*). Together with our data, these reports suggest that the elevated levels of RBD-specific IgG and IgM associate directly with days since onset of symptoms. Future comparisons of our data to antibodies of known specificity, together with the confirmation of viral neutralization in larger cohorts, may provide important insights into the dynamics of antibody responses in different phases of the illness and reveal important differences between antibodies produced in the context of moderate vs. severe disease.

In the memory B cell population, we observed an increased proportion of CD21-CD27- cells in moderate and severe disease, with a parallel decrease in CD21+ CD27+ B cells. CD21-CD27- B cell expansion has been described in other viral infections (*62*–*64*). CD21 (complement receptor type 2, CR2), a co-receptor of the BCR (*65*, *66*), was also significantly down-regulated in severe COVID-19 patients. Activation, binding of complement or TLR stimulation are known to decrease cell surface expression of CD21, and could lead to impaired B cell responses (*67*–*72*). Further studies will be necessary to understand the potential contributions of these CD21 phenotypic and subset alterations to COVID-19 pathogenesis.

T cell activation is typically observed during acute viral infections (*73*–*75*), and as expected (*10*, *13*) we observed increased activation of both CD4+ and CD8+ T cells in severe COVID-19. Remarkably, 18/20 of the top phenotypic parameters distinctive of severe COVID-19 were related to T cell activation. However, T cell activation was very heterogeneous across the severe COVID-19 patients, being equivalent to baseline in some while reaching up to ~25% of memory CD8+ T cells in others. This heterogeneity is relatively unusual compared to the symptomatic phase in other acute infections in humans, such as HIV, EBV, HCMV, HBV, and Ebola, where activation is uniformly detectable but to varying, and sometimes much higher, degrees (*76*–*79*). However, given the degree of lymphopenia observed in the severe COVID-19+ patients, it is possible that activated T cells are migrating to, or sequestered in, the lung in response to the virus (*22*, *80*–*83*), making it unclear if T cell activation is found in other sites as suggested by case study reports (*84*, *85*). We also observed a marked reduction in the frequency of CD161++ CD8+ T cells in donors with severe COVID-19 that was directly associated with APACHE III scores. This subset is composed primarily of mucosal-associated invariant T cells (MAIT) cells (< 95%) (*86*) and a small subset of IL-17 secreting cells (Tc17) (*87*). During viral infections, both MAIT and Tc17 cells can become activated and migrate to infection sites (*87*, *88*). Critically ill COVID-19 individuals were recently shown to have a profound decrease in circulating MAIT cells paralleled with their presence in airways (*36*). As such, the reduction of CD161++/MAIT CD8+ T cells in peripheral blood could be indicative of sequestration in the lungs, potentially exacerbating tissue inflammation.

Many of the immunological characteristics of severe COVID-19 share features of sepsis-associated immune dysregulation, yet others are more specific for an acute viral infection. Decreased expression of CD16 on neutrophils, monocytes, and immature granulocytes and decreased expression of HLA-DR in monocytes has been associated with sepsis and sepsis outcome (*32*, *89*–*91*). However, expansion of plasmablasts and activated T cells is common to typical acute viral infections, not sepsis. Severe COVID-19 is a distinct clinical and immune sepsis subphenotype, and the immune dysregulation may necessitate targeted strategies to effectively manage clinical care. To this end, the immunological analysis strategy that we presented readily differentiated those with severe COVID-19 compared to HD, moderate cases, and recovered cases. Additional studies will still be necessary to understand whether these changes are observed in mild or asymptomatic disease, and the kinetics of their return to baseline levels in recovered individuals. Longitudinal studies to determine whether early detection of the immunological perturbations that we have defined here predicts severe disease trajectory, even when patients exhibit only asymptomatic or mild disease could provide crucial insight into the development of effective therapeutic interventions to ameliorate severe COVID-19.

## MATERIALS AND METHODS

### Study Design

The goal of this study was to perform an unbiased characterization of innate and adaptive immune subsets, associated with humoral responses in SARS-CoV-2 infection resulting in moderate or severe disease, compared to HD and recovered individuals. Recruitment of donors was conducted at the Hospital of the University of Pennsylvania. All participants or their surrogates provided informed consent in accordance with protocols approved by the regional ethical research boards and the Declaration of Helsinki. Sample sizes were based on availability of biological samples rather than a prespecified effect size. Investigators were not blinded to COVID-19 status for safety, but were blinded to disease severity (moderate or severe) while performing experiments. Extended details of cohorts and recruitment are shown in Supplementary Materials and Methods.

## Supplementary Materials

immunology.sciencemag.org/cgi/content/full/5/49/eabd7114/DC1 Materials and Methods Fig. S1. Demographic and clinical information. Fig. S2. Gating strategy used for flow cytometric analyses of immune cell subsets. Fig. S3. Extended innate immune subset characterization and phenotype during COVID-19 infection. Fig. S4. Extended T cell phenotype and activation during COVID-19 infection. Fig. S5. Extended B cell phenotype and total IgG measurements in COVID-19. Fig. S6. Abundance of the top 20 clones in each donor. Fig. S7. Heavy chain variable (VH) gene and CDR3 usage. Fig. S8. Extracellular and intracellular expression of CD16 on NK cells during COVID-19. Fig. S9. Expression of activation markers in CD4+ and CD8+ T cells subsets. Tables S1. Detailed clinical characteristics of individuals with moderate and severe COVID-19. Table S2. Antibody heavy chain gene rearrangement metadata. Table S3. Rotation table extracted from PCA. References (*92*–*100*)
